# Swiss Implementation Science Network (IMPACT): A Crucial Building Block to Strengthen the Swiss Research Pipeline for Real-World Translation

**DOI:** 10.3389/ijph.2021.1604081

**Published:** 2021-04-29

**Authors:** Suzanne R. Dhaini, Juliane Mielke, Thekla Brunkert, Kaspar Wyss, Jürg Utzinger, Sabina De Geest

**Affiliations:** ^1^ Institute of Nursing Science, Department Public Health, Faculty of Medicine, University of Basel, Switzerland; ^2^ University Department of Geriatric Medicine FELIX PLATTER, Basel, Switzerland; ^3^ Swiss Tropical and Public Health Institute, Basel, Switzerland; ^4^ Department of Public Health and Primary Care, Academic Center for Nursing and Midwifery, KU Leuven, Leuven, Belgium

**Keywords:** building block, implementation science, real world translation, IMPACT, swiss research

The failure to translate scientific evidence into practice is well documented in the literature [1]. In 2019, the Swiss Academy of Medical Science described the considerable gap between scientific discoveries and their application in clinical practice as *the valley of death* [2, 3]. Implementation science (IS) has the potential to overcome this gap [2]. Indeed, IS offers a methodological approach to expedite and strengthen the successful translation of safe and effective interventions into real-life settings. In Switzerland, we observe a growing focus on IS. Further developing and strengthening capacities in IS as early as possible in the research pipeline is pivotal to render research more societally relevant [2, 4, 5].

The Swiss Implementation Science Network^1^ (IMPACT) was launched in October 2019. The aims are to (i) showcase the utility of IS by highlighting successful projects; (ii) provide networking and training opportunities within Switzerland and beyond; and (iii) foster funding options for IS in Switzerland [2]. On January 22, 2021, the 1^st^ IMPACT conference was launched, followed by five masterclasses. A total of 237 participants from 25 countries from clinical medicine, epidemiology, nursing, public health among other disciplines participated at the opening event. National and international experts discussed the state of science of IS and illustrated the power of IS for real-world translation of scientific evidence. Four out of seven domains of the “Basel Heptagon of Implementation Science” [2], which highlights specific methodological considerations in IS, were discussed: hybrid designs, theoretical frameworks, contextual analysis, and implementation strategies [2]. Selected examples of successful Swiss IS project were presented including the INTERCARE project [6], whose focus is to develop, implement, and evaluate an interprofessional nurse-led care model in nursing homes to reduce hospitalizations [6].

IS implies a new research paradigm. The two guiding principles are early stakeholder involvement and consistent consideration of real-world application from the conception of an intervention until sustainable implementation. IS builds on clinical research methods, epidemiology, social sciences, system science, and health economics. Successful implementation and sustainability of an evidence-based intervention in real-world settings therefore is team sports. Different disciplines closely collaborate with relevant stakeholders (e.g., patient, families, health insurance, and health policy personnel). The wide variety of disciplines and active stakeholder engagement in IS enriches scientific exchange and exposes the different constituencies to novel methodological approaches with the overarching goal to achieve effective knowledge translation.

In order to further develop IMPACT, we must learn from the experiences of other countries [7, 8]. An online survey was conducted among participants of the 1^st^ IMPACT conference. Internationally, capacity building has been achieved through building a community of implementation scientists with multidisciplinary experts who worked jointly on projects from various backgrounds and disciplines [9]. Accordingly, interdisciplinary alliances fuel IS infrastructures from project-level to research institutions [9]. However, potential barriers to IS exist in view of lack of a common language, methodological challenges, a culture of silo-thinking rather than interdisciplinary collaboration and partnership, and shortage of funding opportunities. Strengthening of IS research infrastructure asks for organizational support, strong leadership, specific funding mechanisms, and resources supported by a clear vision and mission and a strategic plan. IS is a complex endeavor that includes methodological considerations that go beyond typical clinical research (e.g., stakeholder involvement, context analysis, and implementation strategies), and hence, funding schemes need to reflect the budgetary requirements for IS projects. Previous experiences and strategies of international funding agencies can inspire how to best develop IS specific funding schemes.

Training and capacity building come in different sizes, ranging from technical assistance (e.g., reviewing grants, providing scientific advice, etc.) to an increasing number of educational offerings, increasingly in the virtual space (e.g., conferences and webinars) internationally. IS infrastructures need to be supported by good communication (e.g., websites, social media, etc.), in addition to the need to further invest in a common language for IS nationally and internationally.

Our IMPACT survey, specifically assessing needs and priorities for IS infrastructures in Switzerland, reflected the elements listed above and highlighted the way forward for operationalization of IS capacity building in Switzerland. The majority of the participants perceived online training and webinars as most relevant activities, along with the opportunity to exchange with IS experts. The former will be achieved by organizing regular IMPACT webinars, as well as a yearly IMPACT conference. The expressed interest of some participants to more actively engage in IMPACT will be realized by expanding IMPACT’s leadership with IS experts from different Swiss universities. Additionally, we will embed IMPACT into an international IS organization, such as the European Implementation Collaborative.^2^

It is hoped that IMPACT will fuel a vibrant IS community in Switzerland to power the research pipeline for real-world translation thus increasing societal impact of the research money invested. It is a crucial building block in increasing the Swiss IS research capacity, growing the Swiss IS community, while stimulating knowledge circulation nationally and internationally.

## Footnotes

1 ^https://impact-dph.unibas.ch;

2 ^https://implementation.eu
